# The American Dental Association

**Published:** 1894-12

**Authors:** J. M. Walker


### PROCEEDINGS.

### The American Dental Association.

Thirty-fourth Annual Session, at Old Point Comfort, Va., August 7th, 1894.

Reported for the DENTAL REGISTER by Mrs. J. M. Walker.

[*Continued from November Number, page* 564.]

### ETIOLOGY OF DENTAL CARIES—DISCUSSION.

In the discussion of this paper, DR. FRANK ABBOTT said that it was always interesting and instructive to listen to a paper from Dr. S. B. Palmer; that he always brought forward absolutely original ideas and that there was very much in them that should be canvassed carefully by the Association. He had nothing to add to what had been said as to the electric theory of caries. He had placed himself on record upon that question years ago. As to thoroughly drying root-canals, he had long ago given that up as a bad job and does not attempt to dry them in any way, shape or manner. As to the use of essential oils in the canals Dr. Palmer knew what he was talking about in what he said about that. Wash them out with an aqueous solution of bichloride of mercury, 1 in 10,000, and fill. We formerly treated and overtreated and treated to death, but we have learned that filling the canal is the therapeutic means of cutting off the supply of irritating material. When all irritating material is cut off by a proper filling the wound will heal, even with a cyst at the end of the root. Shut off the source of supplies and it will disappear, with the application of a counter-irritant to the gum. Spending time and trouble in trying to dry out a root-canal is of no earthly good, and it is folly to spend hours in the vain attempt.

Dr. J. Y. Crawford: All have seen the condition where there is a lump at the end of the root, which may be either a pus-sac or a nucleated semi-fibrous cyst. If it is a pus-sac what becomes of the pus after the canal is filled?

Dr. Abbott: There is no way of ascertaining the contents of the sac except with the exploring needle—but absorption does take place; without puncture or destroying it in any way, it simply disappears. If pus it may perhaps degenerate and be absorbed.

Dr. Jno. S. Marshall: Many of the gentlemen here have been born into the profession since Dr. Palmer's first papers on this subject were read, but at that time it was my good fortune to be a near neighbor of Dr. Palmer, and it was with great pleasure that I saw going on the experiments which gave rise to the opinions expressed in the paper. In those days men said harsh things about the New Departure, but it has created great changes in modes of practice, and how many now put a pellet of tin at the cervical border of gold filling and save teeth decayed at the cervical margin by filling with amalgam, and the honor is due to Dr. S. B. Palmer. All chemical elements are either positive or negative; all matter the same, even in our foods one is positive and the other negative, and it is for this reason that certain foods are eaten with stronger zest if taken in pairs; the familiar ham and eggs for instance, or roast turkey and cranberry sauce. The saliva connects the two poles. This has been tested and proven by the actual deflection of the needle of the galvanometer. These things have a practical value. We all fill the roots of teeth, but we do it blindly, and not on scientific principles. Try his experiments, and I feel confident that you will get the same results that he does.

Dr. A. W. Harlan wished to ask Dr. Palmer how the essential oils are oxidized in the root of a tooth, and how gutta-percha is oxidized in the root of a tooth. If gutta-percha is an oxidizable substance why was it chosen to cover the great Atlantic Cable? Methods of filling roots of teeth do not properly come under this section. The use of tin at the cervical margins of gold fillings was not introduced by the New Departure men. It was in use for thirty years before the New Departure was born. The experiments of Miller, Black and others prove that this electro-chemical theory of the production of caries has no ground to rest upon. Decay is the result of the agency of micro-organisms through their excretory products. The electro-chemical theory of decay is not proven, but the experiments of Miller will stand until something more definite shall have been demonstrated before scientific bodies.

Dr. Marshall: The theory set forth by Dr. Palmer does not in any way antagonize the experiments and conclusions of Dr. Miller. His theory only relates to what is called secondary decay, the result of introducing metal fillings into teeth of low grade, of soft texture, when from excess of moisture a battery is established in the tooth. He claims that secondary decay is not accounted for in any other way; that it is not always due to faulty manipulation; there is something else that causes that breaking down of tooth structure at the cervical margins of the finest gold fillings. The operation may be as perfect as possible, the gold carefully packed and the filling finely finished, and yet secondary decay occurs—this is the reason, why these failures occur. Dr. Palmer does not claim to have been the first to use tin as described, but I do say that he is the first to give a scientific explanation of its protective value, and he is entitled to that credit.

Dr. A. W. Harlan: That there may be no misconception of my position I claim that the agency that produces decay in the first place produces it in the second, and that the electro-chemical agency has nothing whatever to do with it. If primary decay is due to micro-organism so also is secondary decay. Gold properly inserted in a tooth does not oxidize; it is not soluble.

Dr. J. Y. Crawford: I ask Dr. Harlan, as a therapeutist, which pole attracts acids and which pole attracts alkalies? and I ask him to answer definitely.

Dr. Harlan: I do not know what you seek to obtain.

Dr. Crawford: I have heard an interesting exposition on electricity and it was stated that one pole attracts acids and the other alkalies. Having the negative pole on one side and the positive on the other, medicinal agents are passed through using a battery. Placed on any part of the organism one pole will accumulate acids and the other alkalies. If that is a scientific truth, there is more in the theory of electric influences as a factor in producing caries than we imagine. Dental caries, *per se*, both primary and secondary, is fundamentally the same. There may be many factors in the production of caries but the specific result is the same. They do not act alike, however, under all circumstances and in all environments. If the electrical influence is acid at one pole and alkaline at the other, it may be that it has an important factorage to that effect in dental caries. I believe in making our record as we go along, and I place myself on record here as saying that I believe that the uses of the amalgams have been more hurtful to the human family than beneficial.

Dr. Louis Jack: A few words in explanation of my own inability to accept Dr. Palmer's electro-chemical theory in causing decay of the teeth. It seems to me if that were true that all margins of fillings would have a tendency to decay alike. Because the majority of defects, or secondary decay, occur at the cervical margins, the theory or hypothesis appears to be proven to the minds of a large number of the profession. But Drs. Miller and Black account satisfactorily for the recurrence of decay from the conditions which exist. If we accept the views of Miller and Black we do not need the electro-chemical theory.

Dr. Abbott: One point has not been made clear. If a filling in a tooth is equally perfect in all parts and well finished in every respect, how is it possible for any action to occur between the dentine and the filling, whether electrical or of organisms. The truth is it is all due to imperfect manipulation. There must be something to dissolve the lime salts to start caries. Whether the acids producing this result is due to electricity or to organisms or to some acid substance in the mouth, the final result is the same, so it makes but little practical difference. Dr. Palmer's idea is a good one. It is very feasible that an electric current is passing from a defective filling to the tooth and returning, and that acids are generated. I am not willing to decry the theory for I think there is a great deal in it.

Dr. Bogue: Can Dr. Abbott tell us how long that battery will act?

Dr. Abbott: It will never cease as long as the proper conditions exist.

Dr. Palmer closed the discussion with a further elucidation of the principles involved, and the subject was passed.

Section VI further presented the "Final Report on the Examination of Human Crania," by Dr. J. J. R. Patrick, which at the request of the author was read by title, and also a review of Dr. E. S. Talbot's work on the "Etiology of Deformities of the Head and Face, Jaws and Teeth."

### SECTION VII.

Reported two papers, one on a "New Operation for the Extraction of the Trigeminal Nerve," by Dr. T. W. Brophy, for the relief of neuralgia, which has been successfully performed in three cases. In this operation the foramen is enlarged from above downward, enabling the operator to carry a slender curved drill along the line of the canal, the finger of the left hand recognizing the presence of the drill before it passes through the soft tissues, the contents of the nerve canal being removed more easily than that portion of the dental pulp, which is in the root of the tooth. The operation is especially commendable, in that there is no division of the external tissues, and no hæmorrhage.

In reply to a question, Dr. Brophy said, that the first of these three operations had been made in April, and there had been no recurrence of pain.

Dr. John S. Marshall does not think that sufficient time has elapsed since the operation to pronounce that there will be no recurrence. There may be relief for a year even, and then it may have to be done over again, and perhaps three or four times. As much as three inches of the maxillary nerve has been removed, and yet there will be a reformation unless we reach its peripheral origin. The source of the trouble may be in the ganglion in the brain, or from some spicula of bone pressing on the nerve. Relief in these cases is only temporary, and the same conditions will recur when the nerve tissue is built up. There is no cure. But this is an easier way than the old method, and it does not disfigure the patient, but he would expect hæmorrhage in some cases, necessitating ligating the inferior dental artery.

Dr. Patterson asked what he would do in case that long, slender instrument were to break.

Dr. Brophy: If the instrument is properly tempered it is not likely to happen; but if it did, it would be necessary to remove the maxillary plate, and remove both the instrument and the nerve, but he hoped it would never happen. Dr. Brophy does not think there will ever be a recurrence; the inside of the canal being reamed out by the instrument, bone will be organized from the deposits of plastic matter, and the canal be filled up. There will be no recurrence if the source of trouble is attainable. In one case he found the source of trouble to be, that the nerve had three or four times its normal volume, and appeared as if it had been ligated at the foramen.

A paper by Dr. Wm. N. Morrison, St. Louis, on "Planting Teeth," was next read.

This paper reviewed briefly Dr. Morrison's practice in this line for the past eighteen years, during which time he had not had one unfavorable or dangerous symptom.

Most of the cases reported at the "Centennial," in Philadelphia, have "gone over to join the great majority," but a few are still acting well their part. In his earlier experience he deemed it essential that the tooth to be planted should be *fresh*—not more than twenty-four hours out of the mouth, but since Dr. Younger has demonstrated the value of dry specimens, the operation has been greatly facilitated, though freshly-extracted teeth are preferable. This subject has not yet been thoroughly investigated from the physiological and pathological standpoint. There seems to be an aversion to bringing it up for discussion. When asked why odontoblasts attack promptly the roots of some planted teeth, while others have remained intact for from twelve to fifteen years, no rational or intelligent answer has been given. An unhealthy, negl ted mouth, with the soft tissues in a tumefied condition encourages this retrograde metamorphosis. Physical culture results in the prevention of all classes of microscopic organisms that work destruction in the mouth. Dr. Morrison says: I have accidentally stumbled on the fact that massage treatment with the ball of the finger and the bristles of the toothbrush, over the whole plate of the alveolar process, exerting a friendly friction several times daily, with frequent rinsings with warm water, this will preserve the root of the tooth for a long time, and the patient who thoroughly follows this method will rarely lose a planted tooth.

Dr. Morrison then gave the history of a number of cases of teeth planted from two to fifteen years. One remarkable case of which the early history is on record in the "American Transactions for 1875," is still in good condition, having done good service for nineteen years. It was apparently a desperate case in the beginning. The patient was a lady then forty years of age, of delicate constitution and anæmic habit; the condition of the gum was bad, and receded from this tooth so that a thin broach was easily passed beneath the gum. There was a large decay in the distal surface—had exposed the pulp—having an oxychloride filling, and another decay below this filling. The pulp in the posterior root was dead, but in the anterior root and the body of the tooth it was alive. The patient was anxious to save the tooth, but was not able to stand the necessary operation. The tooth was removed, the roots and crown cavity filled, out of the mouth, deposits of tartar removed, and the tooth returned to place. For three or four days the patient experienced considerable pain, but in a few weeks it became comfortable, and in five months it was the firmest tooth in the arch—which is unbroken. That was the report made in 1875. The tooth was seen recently (1894), and is still in good condition.

Another case reported, was of a tooth which had been replanted by another dentist. A gutta-percha filling had expanded and cracked off the labial face of the crown. The palatine portion and the root being firm, a metal crown was adjusted, but this broke off from too strong occlusion. The implanted root was then removed, and another tooth planted April 26, 1890, which is a magnificent success. In another case a tooth was planted in 1879, which did good service for fourteen years, when a successor was planted in the socket, which is giving satisfaction.

### OPERATIVE DENTISTRY.

The report from Section III. having been deferred, both Chairman and Secretary being absent at the morning session, on Friday, DR. S. G. C. WATKINS stated that no written report being forthcoming from this Section, they could only present some new appliances which the Section deem worthy of consideration.

DR. CHAS. SILL, New York, offers a "Traction Engine," designed for the adjustment of sub-cervical bands on over-jutting teeth, over which shell crowns can be telescoped, avoiding cutting away the enamel in shaping the teeth, as in the ordinary methods.

DR. J. N. CROUSE (for the Dental Protective Supply Co.) offers a new engine bur, which cuts when pressure is straight ahead, as well as with lateral pressure; the blades are also so placed as not to clog. Also a new engine and a new hand-piece, which are worthy of consideration; absorbent pads to be used in the mouth instead of napkins; they are antiseptic, very absorbent, and convenient in size and shape; also a new zinc-phosphate, "Lithos," a slow-setting, dense cement, with which very fine work can be done.

Of this cement, Dr. C. N. Peirce said: This is a pyrophosphate, and on account of the great amount of heat to which it is subjected in the course of manufacture, it is less soluble in the mouth than the cements in general use.

No remarks being made on the subject of Operative Dentistry, Dr. Harlan, Chairman of Section V., "Materia Medica and Therapeutics," announced the report from this Section, which was made by the Secretary, Dr. Geo. E. Hunt. The report was a brief resume of the value of Schrier's preparation of "Kalium Natrium;" of Dr. Callahan's method of opening root canals by the use of Sulphuric Acid; of the value of Pyrozone in the treatment of Pyorrhœa Alveolaris; of Milk of Magnesia in Erosion.

Dr. A. W. Harlan stated that after twelve years service as Chairman of Section V, he now voluntarily retired in favor of Dr. J. S. Cassidy, Covington, Ky. Dr. Harlan reviewed the advances made since 1880 in this branch of dental science. Prior to that date it was included in the curriculum of but one dental college, now it is compulsory in all. He attributes this largely to the work of Section V, of the American Association. Previous to that date, dental treatment was almost entirely mechanical, the few drugs in use being used empirically. Now the internal administration of drugs and the use of antiseptics is an important and essential feature in the treatment of diseases of the antrum, of the dental pulp, and of the oral mucous membrane. All this is evidence of the attainment of advanced knowledge. Among the valuable new remedies mentioned by Dr. Harlan is *alumnol*, an astringent of great value. In the treatment of pyorrhœa pockets, four to seven grains of alumnol, dissolved in water, to which one drop of oil of Ceylon cinnamon is added, injected daily around the roots of the affected teeth for two or three weeks, will cause the pockets to fill up with new growths. This may be preceded by a dilute solution of trichloracetic acid daily for a week, followed persistently by the alumnol injections. In oral catarrhal conditions, nitrate of silver is valuable as a styptic, astringent and stimulant, the objection being the blackening of the tissues. In deep pockets it forms a coagulum before reaching the deepest parts.

The local treatment of pyorrhœa must be based on the theory of micro-organisms, and if the origin is not clearly local, constitutional treatment must be added. The use of pure drinking water in large quantities has a beneficial effect on all mucous surfaces. Gum-massage daily, using 40 grains borax to 1 ounce linolin, to which an essential oil may be added for piquant taste, and a little tannic acid in case the gums bleed, will be found of great assistance.

Dr. Cassidy called attention to a remedy recently introduced, known as "Formalin." It is not a secret nostrum, though given this proprietory name.

Used in root canals it embalms permanently any contents that cannot be removed. Muscular tissue is perfectly cooked by one immersion, forming a homogeneous mass similar to horn. If the yolk of an egg is treated by formalin, no sulphuretted hydrogen is apparent. It is readily absorbed by water, from which the escaping gas is absorbed by solids, as cotton-wool, lint, etc. Absorbed by porous solids, as cakes of pumice, etc., and placed in drawers, it will be valuable in sterilizing instruments, etc., keeping them permanently sterilized. Formalin has been in use too short a time to pronounce positively upon its use in the mouth, but from its known action elsewhere it promises much.

Section V. passed.

Dr. Boice, Secretary of Section I. announced as a supplementary report, a paper from Dr. Wm. N. Morrison, St. Louis, on "Bridges."

The paper was read by title.

### SECTION IV. HISTOLOGY AND MICROSCOPY.

Dr. Frank Abbott, Chairman. No report.

### ADDENDA FOR PATHOLOGY.

Dr. S. H. Guilford: An interesting case in pathology was overlooked when Section VII had the floor. The case was reported to the Odontological Society of Philadelphia by Dr. Gaskell.

Dr. Gaskell: The case is rather unique. The patient presented some two and a half years since, showing a peculiar pinkish spot on the central incisor, showing the same on the labial and palatine walls. The tooth was perfectly sound externally; there had been no irritation and no soreness. As no cause for the appearance was discernable, I advised waiting, thinking it might disappear spontaneously, as it had appeared. But in about six weeks' time the palatal wall crushed in, and the crown was found to have been but a mere shell, the enamel wall of the labial surface being so thin that an instrument could be plainly seen through it. This spring the patient presented with a recurrence of the same thing in the other central incisor. This was shown to Dr. E. C. Kirk. He suggested systemic treatment with iodide of arsenic. I opened into the tooth and found the pulp in normal condition, with perhaps a slight deposit of lime salts, but scarcely perceptible. The pulp was perceptible through the palatine and labial walls, having the appearance of a pink gutta-percha filling inside of very thin enamel walls. After a time there seemed to be a filling up again with a marked decrease in the pink spot. The patient was a college boy about twenty years of age, and in good health. A sample of his blood was examined microscopically and found to be perfectly normal. I would like to have some light thrown on this peculiar phase.

Dr. Jack: My son has had a similar case, and forty years ago I saw a case of the same character. The dentinal substance seems to have been resorbed. The spot in this case was closest to the outer wall of the tooth at the inner or mesial aspect, extending above the margin of the gum. In the more recent case, in my son's practice, he did not consult me, but at once drilled into the tooth, destroyed the pulp, and filled the root with gutta-percha and oxide of zinc. The tooth was perfectly sound and had had no previous treatment. I saw both of the cases described by Dr. Gaskell. The pink spot was very apparent, and as I remember, like the one I saw forty years ago.

Dr. Frank Abbott: This makes five cases of this very peculiar absorption of the inner portion of the tooth, and all reported from Philadelphia. Apparently none have been heard of, from any other place. I have never seen such a case, never heard of anything resembling it. There are but two solutions of the question. Either there must have been some minute external opening into the tooth, through which acids penetrated, creating an irritation of the pulp, causing absorption of the dentine—though how this could possibly have gone on to the extent described is a mystery; and how the lime salts were taken away is another mystery. I do not see how it could have been brought about. The dentine must have been destroyed in a manner similar to the removal of the roots of the temporary teeth when they are absorbed—a dissolving out of the tooth substance. It would seem as though there must have been some irritant in the circulatory apparatus, though if that were the case I do not see why the bones all over the body were not affected and the patient left limp like rubber. That a substance that is harder than any other portion of the body could be dissolved out from the inside of the tooth till the walls were left so thin that they could be seen through, without the presence of any acid to dissolve out the lime salts, with no apparent source of irritation to cause it, is a mystery which is hard to solve, figure it out as best you can.

Dr. John S. Marshall: It can only be explained on the hypothesis of a retrograde metamorphosis of tissue, as the roots of living teeth are sometimes destroyed, the action of osteoclasts in the dental pulp, removing the tissues. The one is as difficult to understand as the other.

Dr. Abbott: By a reversal of the process by which the tooth is built up the lime salts are dislodged and thrown down, dissolved, taken up in the circulation and carried off. I cannot conceive of such a special organ as that called osteoclast—a supposed body, whose peculiar function it is to dissolve lime salts and destroy bone tissue. There are no such things as osteoclasts, but there must have been some irritant. I never heard of these cases except in Philadelphia, but there are many peculiar diseases there never heard of anywhere else.

Dr. Marshall: I stick to the osteoclasts as offering the only explanation. There may have been some preceding shock, perhaps excessive heat or cold to start the irritation, and the subsequent formation of osteoclasts furnishes a satisfactory explanation.

Dr. Abbott: Will Dr. Marshall please explain what an osteoclast is, and where it comes from?

Dr. Marshall: I don't know what an osteoclast is, or where it comes from, but I do know we find what we call osteoclasts.

Dr. Gaskell: I would like to ask what became of the osteoclasts in the case in which treatment was successful?

Dr. Marshall: Osteoclasts are not supposed to work on forever. After they had scooped out the inside of the tooth cementoblasts took up their work and began to fill up that space.

Dr. B. Holly Smith: This reminds me of an anecdote told by Henry W. Grady: Some boys, in a spirit of mischief, pasted together some of the leaves of the Bible in a country church, and when the old preacher read the chapter, he read: "And Noah took unto himself a wife, and she was forty cubits long, and forty cubits broad, well tarred inside and out with pitch." The old man closed the Bible and reverently said: "My brethren, I never observed that passage before, but I believe everything that is inside the cover of this Holy Book. Verily, man is fearfully and wonderfully made!" And so it was with those Philadelphia incisors.

Dr. Palmer: I seldom present anything that I have not demonstrated, but I will offer a suggestion. In discussing a question of this character, we should first consider nature's process in the construction of the living body, and the results of a possible reversal of this process. Circulation being cut off, the current is turned back. No outside exposure is necessary to produce decay below a filling. From irritation of some character, the lime salts are dissolved and carried off, or, on the other hand, lime salts may be deposited and the pulp cavity be filled up. If acids are present it will not fill up, but with a non-irritant, non-conductive substance as gutta-percha, nature may produce currents which will fill up the space. Nature can build up or pull down, according to conditions. As nature deposited the lime salts, nature can re-absorb them, though we cannot say how. We cannot account for this reversal of nature's process.

Dr. Jack: It *is* remarkable that these five cases (I know positively of four) should all have occurred in Philadelphia. The pink appearance was of a higher grade of color than the normal pulp, and the destruction of tissue was very great. In the two cases of Dr. Gaskell's, there was perfect symmetry of the lateral and lingual surfaces of each tooth.

Dr. B. H. Smith: What was the character of the internal surface of the cavity?

Dr. Jack: It was regular and even, with no bay-like excavation, but regular, as if made with a round burring instrument.

Dr. Crawford: I doubt if this could be called hypertrophied pulp. I should call it pulp transformation. The pulp, instead of continuing its work as a tooth builder, became a tooth re-absorber or remover.

Dr. Crawford described a case in which the roots of a right superior sixth-year molar were quite devoid of bony tissue, the living pulp having acted as a tooth destroyer.

Dr. Gaskell had inquired carefully into the precedent history of the cases he described and there had been no irritation, not the slightest soreness. When he removed the pulp the root canal did not show any marked increase in size, though the apical foramen was slightly larger than normal. The pulp was very vascular. It was all removed at once with the aid of cocaine; styptics had been necessary to arrest hæmorrhage.

Dr. Ottofy: Those who invented the theoretical osteoclasts, to account for the peculiar conditions leading to the absorption of the deciduous roots, have never seen one. The theory is defunct, and there was never any necessity for it. The blood stream is a sewer as well as a purveyor. Much useless matter is removed through the blood stream, and there is no reason why dissolved dentine could not be removed in that way. Particle by particle the carbonate and phosphate of lime is brought to the root, and it can be carried away also, in solution, very finely divided, carried off through the blood channel.

Dr. T. T. Moore: I have had a case of this kind, a gentleman 45 or 50 years of age, though it is rare in the human species. There was no decay; the tooth had never had a filling—I had examined the teeth a number of times, but could find nothing wrong. He said he felt impelled to grind and crush his teeth together, and it worried him. I examined most thoroughly, even with the electric light, but could find no external cavity, though I separated the teeth, as he located the trouble between the first and second bicuspids. When he came again one of those teeth had crushed in, and it cut like old cheese. I wrote to the late Dr. Wm. H. Atkinson about it. He said it was a case of liquefaction of the dentine; that it had been decalcified and taken off by the pulp. The same thing recurred in five of his teeth. Nature is busy all the time. She builds up energetically; but when her work is complete she does not remain idle but begins to tear down again. I could find neither systemic nor local cause in this case. It is a singular fact however that the patient could never pare his nails, but had to use a file. There may have been some systemic trouble shown in this relation between the teeth and the nails.

Dr. Abbott: Dr. Gaskell opened the book a little for us when he said that he found the foramen enlarged, and that it was difficult to stop the hæmorrhage. It is fair to presume that through this enlarged foramen there was a larger flow of blood than normal, and the pulp being over-nourished became an irritant liquifying the dentine, the lime salts being taken up and carried away. That one statement opens the way to a reasonable explanation.

Dr. Patterson has had several cases where, though the finest probe failed to reveal any external opening or source of irritation, yet after extraction, closer examination showed such imperfect tooth structure as to prove that the irritation of the pulp came from the outside as surely as though there had been caries. In fact in one case he found quite a large internal cavity, with no opening to the outside that could be detected by the finest instrument. Under a strong glass, however, he found such imperfections in the enamel as to prove that the pulp irritation was directly from outside sources.

Dr. McKellops: I would be glad if any one can tell me how to diagnose an ossified pulp. It is often the cause of intense suffering, when the teeth are, to all appearances, perfect. Extraction offers the only remedy, and then we find an ossified pulp. I will ask some one to tell us how to diagnose this source of suffering.

A patient ten years old recently came to me with a small abrasion of the enamel, near the gingival margin. Another dentist had applied arsenic and the tooth had turned perfectly red. I took it out and found the pulp ossified.

Dr. Crouse: I second the question of Dr. McKellops. Will some one tell us how to diagnose ossified pulps so that we may know when that is the cause of pain. I have a case where I could find no cause for severe suffering, which gives a nervous reflex action all over the mouth, but by tapping on the teeth, I found one tooth a little more sensitive than the others. On that indication, I have removed three teeth in one mouth, and found ossified pulps, and I now suspect another.

Dr. Gaskell: It is better to drill into the tooth and find out. Extracting the tooth is like cutting off a man's head to save his life.

Dr. Brophy: With the aid of an electric incandescent light, in a darkened room, you can nearly always determine whether the pulp is calcified or not. An unusual degree of opacity is seen in a tooth so affected.

Dr Jno. S. Marshall: This is one of the most difficult things to diagnose. We can only conclude, if there is no other possible cause this must be it. Unless we resort to the drill as suggested, but we do not always know which tooth to drill into. The only way is by the process of exclusion. If we cannot find any other symptom it must be ossification. In young persons, if there is a spicula, the pain will be increased by anything which increases blood pressure, as by rapid walking, bending over, causing the blood to run to the head, anything causing extra pressure on the arterial system—causing expansion of the pulp. This may help you some times.

Dr. Abbott: What causes ossification of the pulp? Why does it occur? What is the origin of the trouble? Let us all think this subject over during the coming year, and talk it over again at our next meeting.

Dr. McKellops protested against passing the subject of Operative Dentistry simply because the Section offers no report. He said: "I would like to ask what we, as a profession, make our living from?" By Operative Dentistry, at least ninety per cent. of it, and yet we have no report and no discussion of the subject in which we are practically most interested—that by which we support our families. Give these young men the benefit of your experience on this, the most practical part of dentistry—that by which they must make their living.

The subject was passed, the time set apart for elections having arrived. Asbury Park was selected as the next place of meeting, on the first ballot.

OFFICERS ELECTED.

J. Y. Crawford, Nashville, Tennessee, President.

S. G. C. Watkins, Mont Clair, N. J., First Vice-President.

Thos. Fillebrown, Boston, Second Vice-President.

Geo. H. Cushing, Chicago, Recording Secretary.

Emma Eames Chase, St. Louis, Mo., Corresponding Secretary.

Henry W. Morgan, Nashville, Tennessee, Treasurer.

C. N. Peirce, H. A. Smith, T. S. Waters, L. Ottofy, Executive Committee.

DENTAL PROTECTIVE ASSOCIATION.

At the request of Dr. J. N. Crouse, a committee of three from the American Dental Association and two from the Southern Dental Association, were appointed to examine the books and papers of the Dental Protective Association.

The committee consisted of Drs. H. A. Smith, Louis Jack, C. E. Esterly and Drs. R. R. Freeman and V. E. Turner, reported at a subsequent session that they had examined all of the books and documents submitted by Dr. Crouse, and found the funds fully accounted for, and the affairs of the Association administered with discrimination and economy.

THE DISCOVERY OF ANÆSTHESIA.

On a resolution offered by Dr. J. D. Thomas, the following committee was appointed to take into consideration and report suitable measures for the celebration of the fiftieth anniversary of the discovery of anæsthesia by nitrous oxide, by Horace Wells. The committee consisting of Drs. J. D. Thomas, Philadelphia; Thos. Fillebrown, Boston; J. Taft, Cincinnati; Jas. McManus, Hartford; H. W. Morgan, Nashville; W. C. Barrett, Buffalo; A. W. Harlan, Chicago; H. J. McKellops, St. Louis; and T. E. Weeks, Minneapolis, reported a series of resolutions, enumerating appropriate features for the celebration, including two papers: one, giving the "History of the Discovery of Anæsthesia," to be prepared by Dr. Fillebrown, and one on "The Application of Anæsthesia to Surgery, and its Benefits to Mankind," which Dr. J. E. Garretson would be invited to prepare. Also that a banquet be given and prominent gentlemen to be invited to make addresses. All of these papers to be preserved in a memorial volume.

Philadelphia was selected as the place for the celebration.

An organization committee of nine, an executive committee of eleven, and a general committee of fifty were recommended in the report which was adopted.

Dr. Jas. McManus offered the following resolution, which was adopted:

WHEREAS, Papers printed in the magazines of the day are calculated to mislead the public as to the facts in regard to the discovery of anæsthesia;

*Resolved*, That the American Dental Association places itself on record, as it did in 1864, by affirming that the late Dr. Horace Wells, of Hartford, Connecticut, is entitled to the honor and credit of the discovery. The justice of Dr. Wells' claim was established by an official investigation before a committee of Congress. The testimony taken at this investigation is printed in a work on "Modern Anæsthesia," published in 1859; and in 1867 by the late Hon. Truman Smith, U. S. Senator from Connecticut.

### UNIFICATION OF DENTAL LAWS.

Drs. B. Holly Smith, A. W. Harlan and J. Y. Crawford were appointed a committee to confer with similar committees from other dental societies, in an effort to secure unification of the dental laws of the different States, and especially in regard to registration and license to practice dentistry.

### CENSUS CLASSIFICATION OF DENTISTS.

Dr. A. Boice called attention to the statements made in the transactions of 1892, in regard to the fight made on the census question. A great deal of work was done by men whose names are not mentioned, and who are entitled to credit in the matter. Drs. A. Boice, W. S. Twilley and C. A. Meeker, were appointed a committee to prepare a correct history of the movement, to report at the next meeting.

### FINANCE COMMITTEE WORLD'S COLUMBIAN DENTAL CONGRESS.

At the request of Dr. Jno. S. Marshall, Treasurer, a committee of two was appointed to examine the accounts of the Treasurer of the Executive Committee of the World's Columbian Dental Congress.

Drs. M. W. Foster and H. B. Noble, the committee appointed, reported that they had examined the accounts and vouchers of Dr. Marshall and found everything correct.

Dr. Marshall presented a detailed statement, showing over $14,000 received from foreign governments, State and Local Dental Societies, etc. Also vouchers for expenditures, showing, with bills paid and contracted, a deficit of about $1,000.

The Association voted an additional donation of $500, and before the close of the meeting, Dr. Marshall had raised $260 of the remaining $500, and hoped to receive the full amount, in order to pay for the Transactions now being printed.

### NECROLOGY.

The Committee on Necrology, Drs. J. Taft, C. N. Peirce, H. J. McKellops, Louis Jack, F. Abbott, reported suitable resolutions in memory of those who have fallen from the ranks during the past two years. The long list includes :

Drs. Geo. Watt, Xenia, Ohio.
W. W. Allport, Chicago, Ill.
W. H. Eames, St. Louis, Mo.
A. W. Rawls, Lexington, Ky.

R. B. Winder, Baltimore, Md.
Edgar Park, St. Louis, Mo.
Alonzo B. Beale, Philadelphia, Pa.
Ambrose Lawrence, Boston, Mass.
J. Smith Dodge, New York, N. Y.
W. C. Wardlaw, Augusta, Ga.
J. H. Smith, New Haven, Conn.
M. H. Dodge, New York, N. Y.
Fred A. Levy, Orange, N. J.
G. W. McIlhaney, Columbus, Ga.
G. A. Wells, Indianapolis, Ind.

### GENERAL ADDRESSES.

Dr. A. W. Harlan introduced a resolution, providing for two general addresses on some subjects of universal interest, to be delivered on the second and third days, respectively, of the annual sessions. The third division of the Executive Committee to name the subjects, and the first division to select the essayists.

The resolution was adopted, and Dr. A. W. Harlan was selected to deliver an address on "Antiseptic Surgery," and Dr. Louis Jack, one on "The Business of Dentistry."

### COMMITTEE ON STATE AND LOCAL SOCIETIES

reported that in the discharge of their functions, they had formulated a series of questions to serve as topics for discussion by the Local and State Societies throughout the country. An important part of the plan, was the anticipated reception of reports of these discussions, but only one such report has been received by the committee. They are still of the opinion that the scheme has its value, the dental journals showing that interest in the plan has been wide-spread, and that the topics suggested have furnished abundant material for study and discussion.

On motion, the committee was continued, with authority to promulgate another series of questions for discussion by State and Local Societies during the coming year.

CONSOLIDATION OF SOUTHERN AND AMERICAN DENTAL ASSOCIATIONS.

Dr. Thos. Fillebrown said that a certain old farmer, being asked the best time to plant corn, replied: "When the ground is ready." He believes the ground is now ready for planting seed which will germinate, grow and bear fruit through closer union among the members of the dental profession, resulting in more rapid, scientific and professional advancement.

He moved the appointment of a committee of five to confer with the Southern Dental Association in regard to combining the two Associations in one National Association.

Drs. Thos. Fillebrown, B. Holly Smith, Louis Jack, J. Y. Crawford and J. N. Crouse were appointed on this committee.

The report of the

COMMITTEE ON REVISION OF THE CONSTITUTION

was deferred for a year, and the committee continued, in view of the changes which will be necessitated, should the proposed consolidation be effected.

TERMINOLOGY.

Dr. B. Holly Smith moved the appointment of a permanent committee, whose duty it shall be to prepare a system of terminology, to report progress, and be continued from year to year. The motion was adopted and the following committee appointed: Drs. S. H. Guilford, L. Ottofy, M. L. Rhein, Thos. E. Weeks, C. L. Goddard, L. D. Shepard, D. R. Stubblefield, Grant Molyneux, A. H. Thompson.

PYORRHŒA ALVEOLARIS.

On motion of Dr. J. Taft, a committee of five was appointed for the investigation of the disease commonly called "Pyorrhœa Alveolaris," with the view of ascertaining the etiology, pathology and correct modes of treatment of this disease. The committee was authorized to draw on the treasury for a sum, not to exceed seventy-five dollars, for the necessary expenses of this investigation. Drs. C. N. Peirce, John S. Marshall, E. C. Kirk, M. L. Rhein and J. Taft, were appointed on this committee.

Association Adjourned.

